# Serum anti-CFL1, anti-EZR, and anti-CYPA autoantibody as diagnostic markers in ovarian cancer

**DOI:** 10.1038/s41598-024-60544-2

**Published:** 2024-04-29

**Authors:** Yifan Cheng, Qing Li, Guiying Sun, Tiandong Li, Yuanlin Zou, Hua Ye, Keyan Wang, Jianxiang Shi, Peng Wang

**Affiliations:** 1 College of Public Health, Zhengzhou University, Zhengzhou, 450001 Henan Province China; 2 Henan Key Laboratory of Tumor Epidemiology and State Key Laboratory of Esophageal Cancer Prevention and Treatment, Zhengzhou University, Zhengzhou, 450052 Henan Province China; 3 Henan Institute of Medical and Pharmaceutical Sciences, Zhengzhou University, Zhengzhou, 450052 Henan Province China; 4 School of Basic Medical Sciences, Academy of Medical Science, Zhengzhou University, Zhengzhou, 450052 Henan China

**Keywords:** Diagnostic marker, Ovarian cancer, Tumor-associated antigens, Tumor-associated autoantibodies, Biomarkers, Oncology, Gynaecological cancer, Tumour biomarkers, Tumour immunology

## Abstract

The purpose of this study was to identify novel autoantibodies against tumor-associated antigens (TAAs) and explore a diagnostic panel for Ovarian cancer (OC). Enzyme-linked immunosorbent assay was used to detect the expression of five anti-TAA autoantibodies in the discovery (70 OC and 70 normal controls) and validation cohorts (128 OC and 128 normal controls). Machine learning methods were used to construct a diagnostic panel. Serum samples from 81 patients with benign ovarian disease were used to identify the specificity of anti-TAA autoantibodies for OC. In both the discovery and validation cohorts, the expression of anti-CFL1, anti-EZR, anti-CYPA, and anti-PFN1 was higher in patients with OC than that in normal controls. The area under the receiver operating characteristic curve, sensitivity, and specificity of the panel containing anti-CFL1, anti-EZR, and anti-CYPA were 0.762, 55.56%, and 81.31%. The panel identified 53.06%, 53.33%, and 51.11% of CA125 negative, HE4 negative and the Risk of Ovarian Malignancy Algorithm negative OC patients, respectively. The combination of the three anti-TAA autoantibodies can serve as a favorable diagnostic tool for OC and has the potential to be a complementary biomarker for CA125 and HE4 in the diagnosis of ovarian cancer.

## Introduction

Ovarian cancer (OC) is one of the three most common malignancies of the female reproductive system and ranks eighth in morbidity and mortality among all cancers in women^1^. OC’s lack of specific symptoms, an effective screening, and diagnostic techniques make it difficult to diagnose at an early stage, resulting in poor survival due to metastasis^2^. However, when the tumor is diagnosed at the early stage, the survival rate can increase to about 90%^3^. Currently, the most commonly used serological biomarkers for OC screening and diagnosis are tumor-associated antigens (CA125) and human epididymis protein 4 (HE4), but due to their insensitivity to early OC detection and high false positive rates, their predictive utility for OC screening and diagnosis remains suboptimal^4–6^. Therefore, there is still a need to develop new biomarkers to improve early diagnosis and therapeutic efficacy of OC.

During the occurrence and progression of tumors, tumor-associated antigens (TAAs) may be produced due to abnormal expression, mutation or post-translational modification of proteins or other reasons. And these TAAs will trigger the immune response to generate anti-TAA autoantibodies^7^. As a potential source of diagnostic biomarkers, anti-TAA autoantibodies show more advantages than corresponding TAAs: (a) circulating anti-TAA autoantibodies exhibit greater stability than corresponding TAAs over time^8^; (b) the immune response to TAAs results in an amplified signal, such that anti-TAA autoantibodies may be detectable easier and earlier than the TAAs themselves^9,10^. As demonstrated by many studies, anti-TAA autoantibodies have been found to be a promising cancer detection tool^11,12^, and they have also shown diagnostic potential in the female reproductive system malignancies^13,14^.

Literature review found Cofilin 1 (CFL1), Ezrin (EZR), Cyclophilin-A (CYPA), Profilin 1 (PFN1), Napsin A (NAPSA) have been found to be associated with the development and progression of OC^15–19^, and are potential TAAs for ovarian cancer. Among them, the anti-TAA autoantibodies of CFL1, EZR, CYPA have been shown diagnostic potential in esophageal cancer, pancreatic cancer, and breast cancer^20–24^. Both CFL1 and PFN1 are actin-binding proteins, showing potential correlation function^25^. NAPSA was found to be strongly expressed in some subgroups of OC tissue, but not significantly expressed in the normal ovarian tissue^19,26^. In view of the fact that the existence of anti-TAA autoantibodies of the above five TAAs in OC has not been explored, this study tested the expression levels of the serum anti-TAA autoantibodies of the above five indicators, evaluated its value in the serological diagnosis of OC, and established a diagnostic panel with a group of anti-TAA autoantibodies for OC diagnosis.

## Materials and methods

### Study samples

Three independent cohorts consisting of 198 OC, 198 normal controls (NC) and 81 benign disease (BD) samples were included in this study. The discovery cohort included 70 OC patients and 70 NC. The validation cohort contained 128 OC patients and 128 NC. The BD validation cohort consisted of 81 BD patients, as well as 81 OC and 81 NC samples selected from all samples according to the matching principle and simple random sampling. All samples between groups in each cohort were matched using frequency matching method by age (± 3 years) and sex.

The OC and BD samples were obtained from two Three-A hospitals in Henan Province from July 2017 to December 2018. Clinical, surgical and laboratory examination information was obtained through electronic medical records. All patients were pathologically and histologically diagnosed as OC and had no other malignancies or immune diseases. The clinical stage was determined according to the International Federation of Gynecology and Obstetrics (FIGO) ovarian cancer classification guidelines^27^. The NC samples were obtained from the Biological Specimen Bank of Henan Key Laboratory of Tumor Epidemiology. All NC samples were taken from healthy individuals without malignant or immune-related diseases. This study was executed in accordance with the Declaration of Helsinki, and was approved by the Ethics Committee of Zhengzhou University. The informed consent was obtained from all participants.

The blood samples were centrifuged at 1500*g* for 10 min after collection and were stored at − 80 °C. All procedures were conducted in adherence to relevant guidelines and regulations.

### Enzyme-linked immunosorbent assay (ELISA)

The recombinant proteins (NAPSA, CYPA, CFL1, EZR, PFN1) were obtained from Cloud-Clone Corp. (Wuhan, China). Each protein was diluted to the optimal usable concentration obtained by pretest for ELISA detection. The detailed steps are as described earlier^28^. It is noteworthy that the same 4 OC and 4 NC samples were added to each plate in order to normalize different plates. The resulting coefficient of variation was less than 15%. Finally, attained the optical density (OD) at 450 and 620 nm by reading it with PerkinElmer’s Multilabel Plate Reader.

### Development of panel for ovarian cancer detection

The model was trained with validation cohort containing 256 samples and validated in the discovery cohort containing 140 samples. A total of seven classifiers, including a enter-logistic regression (ELR), a forward logistic regression (FLR), a random forest (RF), a support vector machine (SVM), a gradient boosting decision tree (GBM), a Naive Bayes (NB), and a neural network (NN) were constructed. Tenfold cross-validation was applied reiteratively 10 times to strengthen the robustness of predictions by the models. Three clinical indicators, CA125, HE4, and risk of ovarian malignancy algorithm (ROMA), obtained through electronic medical records, were used to compare with the panel. The cutoff value of CA125 was 35 u/ml. The cutoff values of HE4 in premenopausal and postmenopausal patients were 68.96 and 114.90 pmol/l, respectively. ROMA was calculated as described above^29^.

### Statistical analysis

The IBM SPSS Statistics 25, GraphPad Prism 8.0, and R version 4.2.2. were applied in this study. Mann–Whitney U Test and Chisquare test were performed to compare the differences of anti-TAA autoantibodies levels between the two groups. When comparing the positive rates of different diagnostic indicators, McNemar’s test is used. The receiver operating characteristics (ROC) curve analysis was used to evaluate the diagnostic value. The sensitivity, specificity, Youden index (YI), accuracy, positive predictive value (PPV), and negative predictive value (NPV), positive likelihood ratio (+ LR), negative likelihood ratio (− LR) were calculated to evaluate the validity and reliability of the diagnostic biomarkers. *P* < 0.05 was defined to be significant.

## Results

### Study design

**Figure 1:**
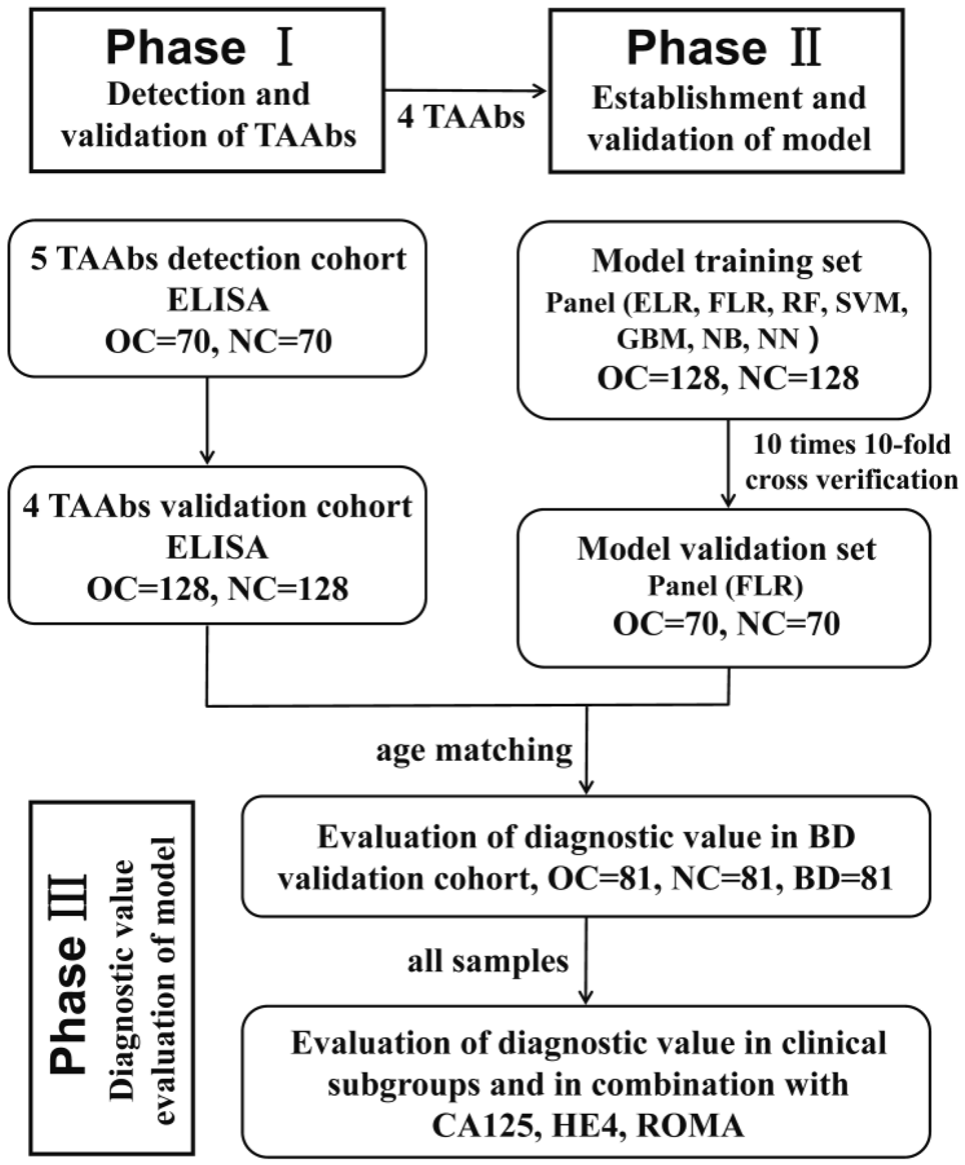
Study design. BD, benign disease; CA125, tumor-associated antigens; ELR, enter-logistic regression; ELISA, Enzyme-linked immunosorbent assay; FLR, forward logistic regression; GBM, HE4, human epididymis protein 4; gradient boosting decision tree; NB, Naive Bayes; NC, normal controls; NN, neural network; OC, ovarian cancer; RF, random forest; ROMA, risk of ovarian malignancy algorithm; SVM, support vector machine; TAAbs, anti-tumor-associated antigen autoantibodies.

The characteristics of all samples are shown in Table 1. This study involved three stages (Fig. 1): (a) Candidate anti-TAA autoantibodies detection was performed on 140 serum samples from the discovery cohort by ELISA, and validated on 256 serum samples from the validation cohort; (b) based on the data of the validation cohort, machine learning methods were used to explore the optimal diagnostic model, and the data from the discovery cohort was used for validation; (c) based on the data of the BD validation cohort, the panel was evaluated for differential diagnosis ability, performance in clinical subgroups, and performance in combination with CA125, HE4, and ROMA.

**Table 1:** Characteristics of ovarian cancer (OC) patients in the discovery and validation cohort.

Variables	Discovery cohort		Validation cohort		BD validation cohort		
	OC (%)	NC (%)	OC (%)	NC (%)	OC (%)	NC (%)	BD (%)
Number	70	70	128	128	81	81	81
Female	70 (100)	70 (100)	128 (100)	128 (100)	81 (100)	81 (100)	81 (100)
Age, years*							
Mean ± SD	53.3 ± 11.4	52.2 ± 11.8	52.7 ± 9.6	51.1 ± 9.8	45.9 ± 10.1	45.6 ± 10.1	44.2 ± 10.3
Range	19–73	20–76	22–81	23–83	19–68	20–69	20–68
FIGO							
I	9 (12.8)		16 (12.5)				
II	9 (12.8)		16 (12.5)				
III	33 (47.1)		51 (39.8)				
IV	12 (17.1)		32 (25.0)				
Unknown	7 (10.0)		13 (10.1)				
Histologic type							
Epithelial tumor	54 (77.1)		107 (83.5)				
Sexual cord interstitial tumor	2 (2.8)		7 (5.4)				
Germ cell tumor	4 (5.7)		4 (3.0)				
Unknown	10 (14.2)		10 (7.8)				
Lymph node metastasis							
Yes	43 (61.4)		76 (59.3)				
No	22 (31.4)		36 (28.1)				
Unknown	5 (7.1)		16 (12.5)				
Distant metastasis							
Yes	12 (17.1)		32 (25.0)				
No	54 (77.1)		85 (66.4)				
Unknown	4 (5.7)		11 (8.5)				
Menopause							
Yes	56 (80.0)		101 (78.9)				
No	10 (14.2)		21 (16.4)				
Unknown	4 (5.7)		6 (4.6)				

FIGO, International Federation of Gynecology and Obstetrics; OC, Ovarian cancer; NC, normal control.

*Age matching between the groups in the cohort was ± 3, and there was no difference among all groups by T-test.

### Detection and validation of tumor-associated autoantibodies for ovarian cancer detection

**Figure 2:**
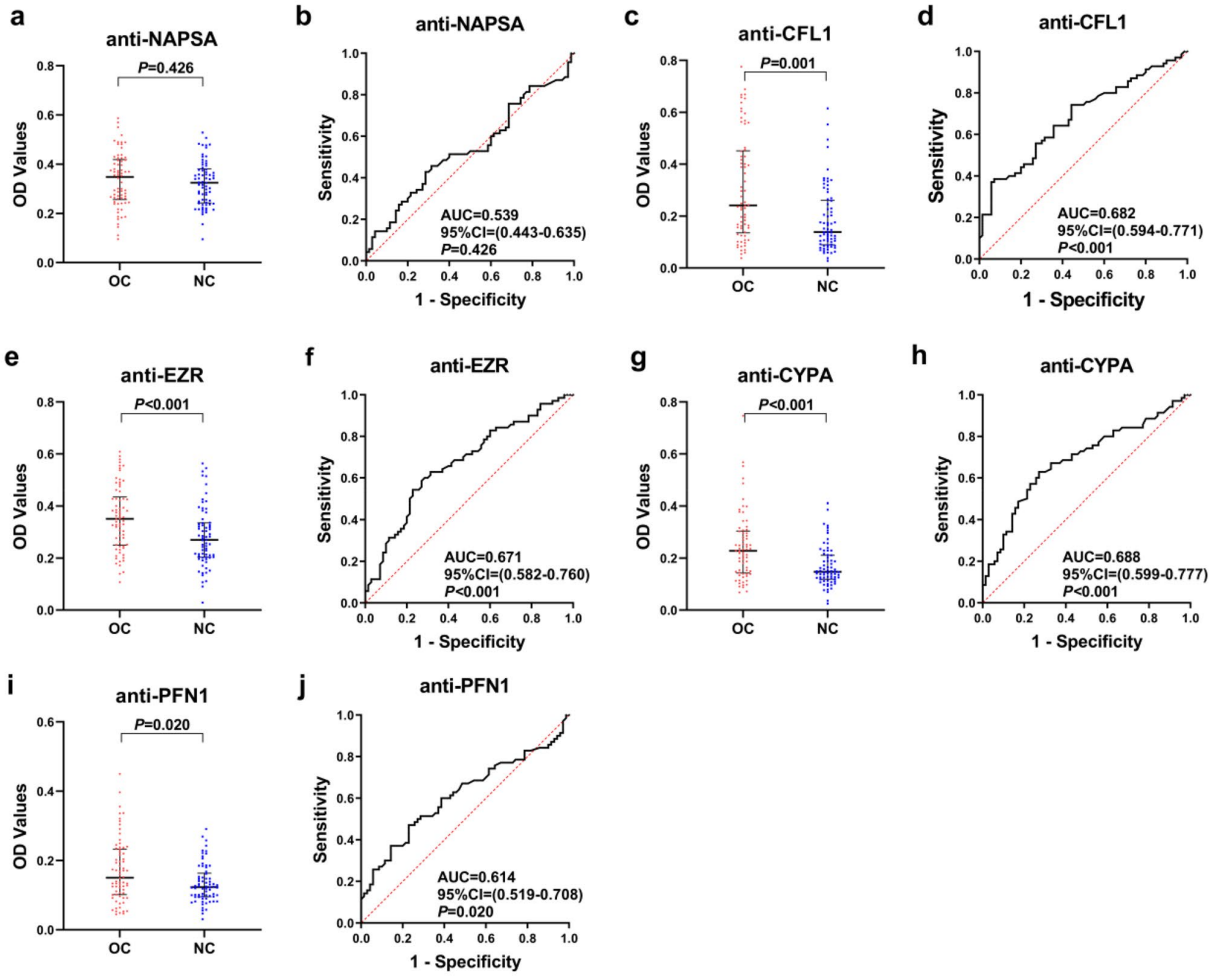
Performance of the five anti-TAA autoantibodies in the discovery cohort. (**a**, **c**, **e**, **g**, **i**) Scatter plots and (**b**, **d**, **f**, **h**, **j**) receiver operating characteristic curve (ROC) for five anti-TAA autoantibodies in the discovery cohort. The longest line means median, and the 25th and 75th percentiles are represented by shorter lines.

**Figure 3:**
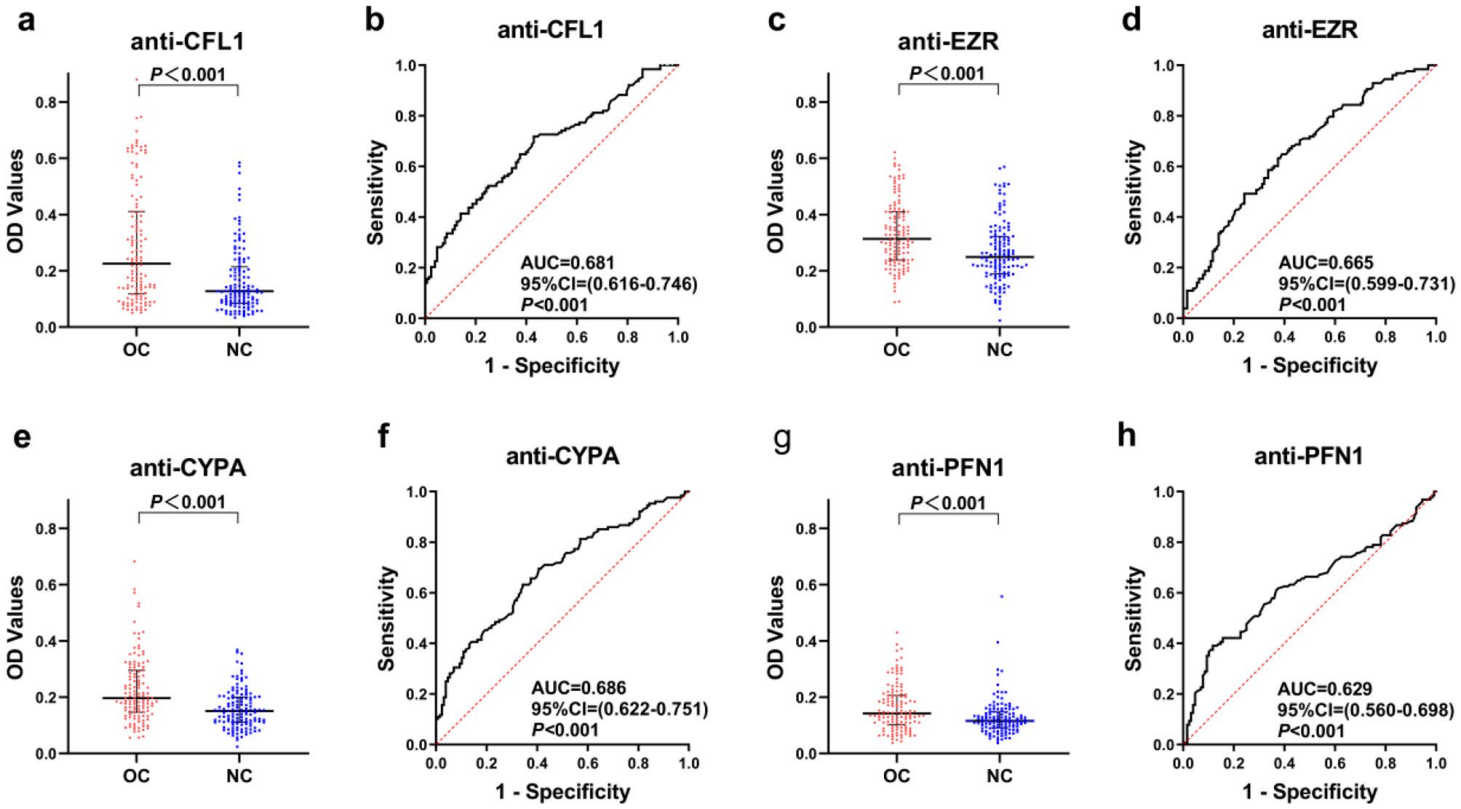
Performance of the four anti-TAA autoantibodies in the validation cohort. (**a**, **c**, **e**, **g**) Scatter plots and (**b**, **d**, **f**, **h**) receiver operating characteristic curve (ROC) for four anti-TAA autoantibodies in the validation cohort. The longest line means median, and the 25th and 75th percentiles are represented by shorter lines.

In this phase, five anti-TAA autoantibodies (anti-NAPSA, anti-CFL1, anti-EZR, anti-CYPA, anti-PFN1) were detected by ELISA in the discovery cohort. Their expression in serum and ROC curves are presented in supplementary table 1 and Fig. 2. The expressions of four anti-TAA autoantibodies (anti-CFL1, anti-EZR, anti-CYPA and anti-PFN1) in the OC were significantly higher than those in the NC (*P* < 0.05). The range of AUC of single anti-TAA autoantibody was 0.539–0.688. Next, four significantly different anti-TAA autoantibodies were further confirmed in the validation cohort. Their expression in serum and ROC curves are presented in supplementary table 1 and Fig. 3. The results also confirmed that the expression of four anti-TAA autoantibodies in the OC were significantly higher than those in the NC (*P* < 0.05). The range of AUC of four anti-TAA autoantibodies was 0.629–0.686. The positive rate and diagnostic value of each anti-TAA autoantibody are shown in supplementary table 2.

### Establishment and validation of a panel with anti-TAA autoantibodies for ovarian cancer detection

**Figure 4:**
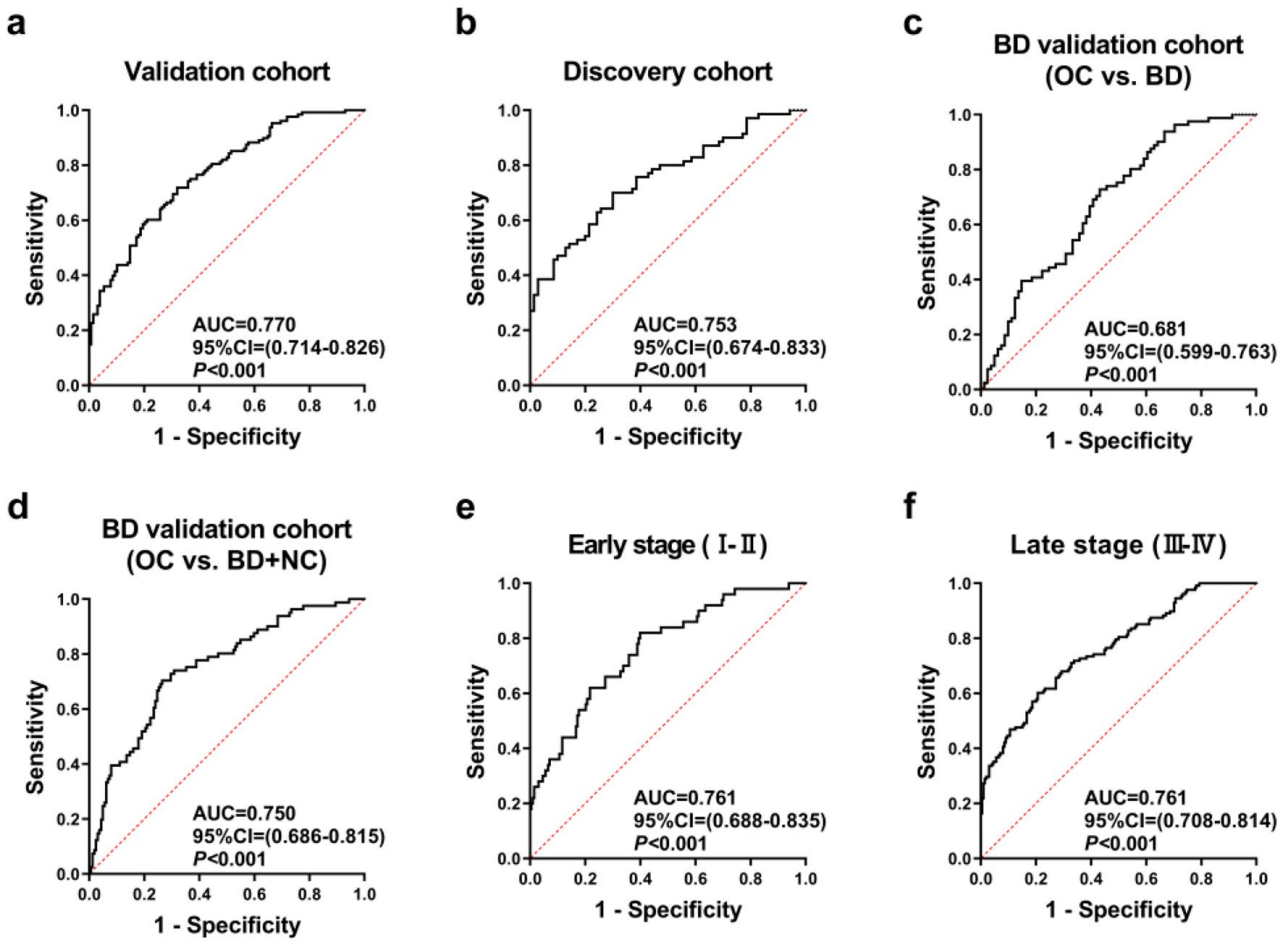
Performance of the panel with three anti-TAA autoantibodies to detect ovarian cancer. (**a**) Receiver operating characteristic (ROC) curves of the panel (CFL1, EZR, CYPA) constructed by forward logistic regression method in validation cohort, (**b**) discovery cohort, (**c**) BD validation cohort (OC vs. BD), (**d**) BD validation cohort (OC vs. BD + NC), (**e**) Early stage (I–II) and (**f**) Late stage (III–IV). OC, Ovarian cancer; NC, normal control; BD, benign disease.

The average AUC obtained after tenfold cross-validation of the seven models ranged from 0.694 to 0.759, as shown in supplementary table 3. Since the panel trained by forward logistic regression method has the highest AUC and the lowest number of indicators, we choose this panel as the optimal panel. The predicted possibility of diagnosis as OC was PRE (P = OC, 3 anti-TAA autoantibodies) = 1/(1 + EXP (−(− 3.399 + 4.512 × anti-CFL1 + 3.997 × anti-EZR + 6.71 × anti-CYPA))). The ROC curve and diagnostic value of this model in the training set (validation cohort) and verification set (discovery cohort) are shown in Table 2 and Fig. 4. The AUC is 0.770 (95% CI 0.714–0.826) and 0.753 (95% CI 0.674–0.833), sensitivity is 58.59% and 51.43%, specificity is 80.47% and 84.29%.

**Table 2:** Diagnostic value of the anti-TAA autoantibodies panel of OC patients in different cohorts.

Cohort	Positive (%)		Se (%)	Sp (%)	YI	Accuracy (%)	PPV (%)	NPV (%)	+ LR	− LR
	BC	NC								
Training set (Validation cohort)	75 (64.20)	25 (21.43)	58.59	80.47	0.39	69.53	75.00	66.02	3.00	0.51
Validation set (Discovery cohort)	41 (58.57)	15 (21.43)	51.43	84.29	0.36	67.86	76.60	63.44	3.27	0.58
BD validation cohort (OC vs.BD)	52 (64.20)	32 (39.51)	39.51	83.95	0.23	61.73	71.11	58.12	2.46	0.72
BD validation cohort (OC vs.BD + NC)	52 (64.20)	40 (49.38)	51.85	80.86	0.33	71.19	57.53	77.06	2.71	0.60

+ LR, positive likelihood ratio; − LR, negative likelihood ratio; NPV, negative predictive value; PPV, positive predictive value; Se, sensitivity; Sp, specificity; YI, Youden index;

### Specificity of the panel in the diagnosis of ovarian cancer among ovarian diseases

**Figure 5:**
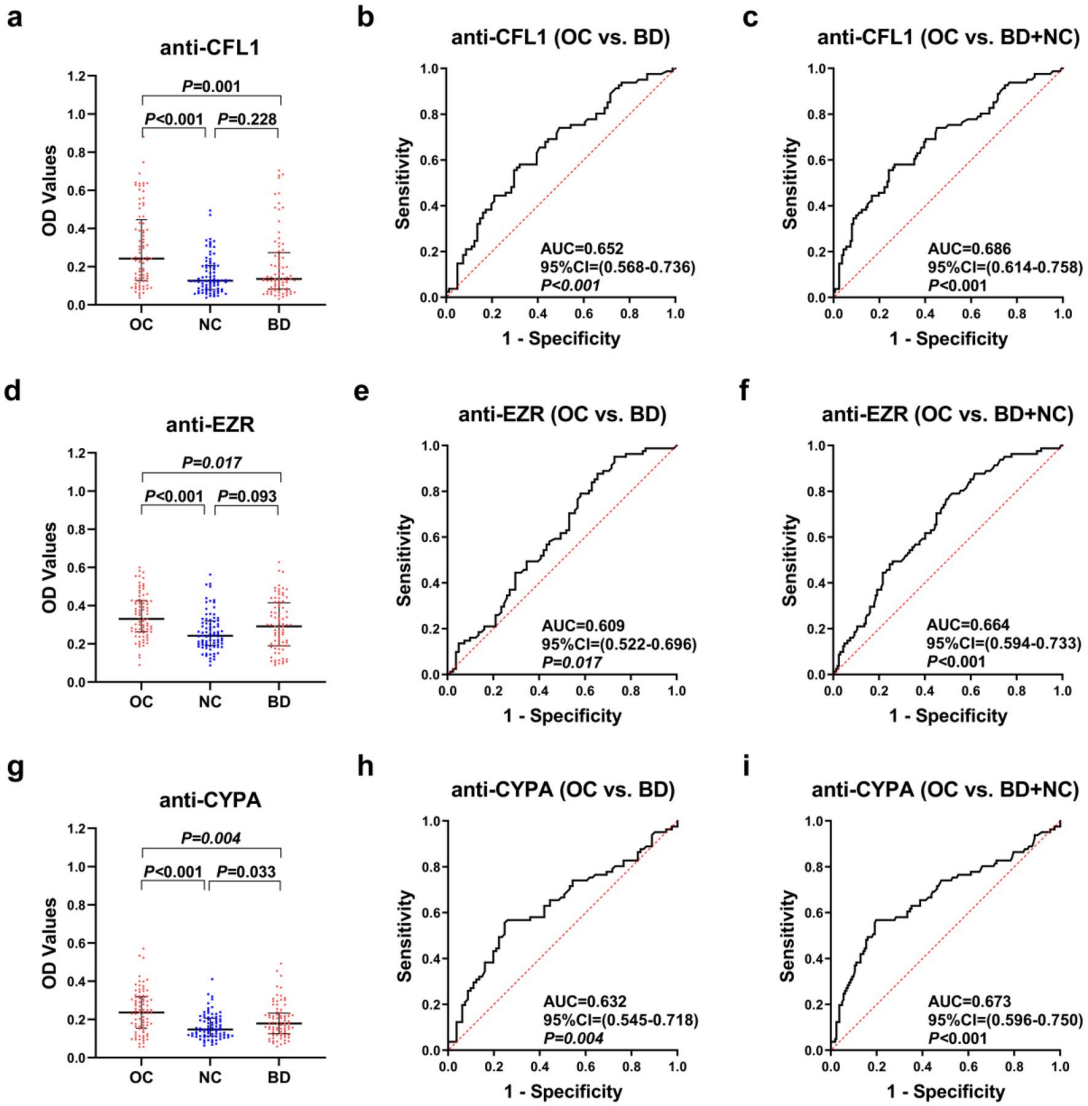
Performance of the three anti-TAA autoantibodies in BD validation cohort. (**a**, **d**, **g**) Scatter plots and (**b**, **c, e**, **f**, **h**, **i**) receiver operating characteristic curve (ROC) for three anti-TAA autoantibodies between three groups. The longest line means median, and the 25th and 75th percentiles are represented by shorter lines.

In order to explore whether the constructed model can distinguish OC from benign ovarian disease, anti-TAA autoantibodies (anti-CFL1, anti-EZR, and anti-CYPA) were detected in the BD validation cohort. As shown in Table 2 and Fig. 4, the model we established has an AUC of 0.681 (95% CI 0.599–0.763), a sensitivity of 39.51%, and a specificity of 83.95%, when distinguishing OC from BD. When distinguishing OC from BD and NC, the model we established has an AUC of 0.750 (95% CI 0.686–0.815), a sensitivity of 51.85%, and a specificity of 80.86%. When analyzing each indicator individually, as shown in Fig. 5, the levels of each anti-TAA autoantibody in OC group were significantly higher than those in the BD group and the NC group, and there was no difference between BD group and NC group except anti-CFL1. When these anti-TAA autoantibodies were used to distinguish between OC and BD, the AUC were 0.625, 0.609, 0.632, respectively. As for distinguishing both BD and NC, the AUC were 0.686, 0.664, 0.673. The positive rate and diagnostic value of three anti-TAA autoantibodies are shown in supplementary table 4.

### Performance of the panel for ovarian cancer subgroups

We evaluated the diagnostic value of the panel in different clinical subgroups (Supplementary table 5). The panel provided an AUC of 0.761 with the sensitivity of 54.00% and the specificity of 82.32% in early detection of OC (stage I–II), and there was no significant difference compared with that in late detection of OC (stage III–IV) (*P* = 0.995), as shown in Fig. 4. Besides, there was no significant difference in the diagnostic value of the panel to distinguish the OC patients of different clinical subgroups of age, histological types, the presence of lymph node metastasis, the presence of distant metastasis and the presence of menopause (*P* > 0.05) (Supplementary table 5).

### Comparison of diagnostic value of panel with CA125 and HE4

In this study, serum expression levels of CA124 and HE4 could be determined in 134 and 89 of 198 patients with OC, respectively. In 2 cases, ROMA values could not be calculated because the presence of menopause was not clear.

As shown in Supplementary table 5, there was no significant difference in the diagnostic value of the panel in OC patients across different clinical indicator groups (*P* > 0.05). As for the positive rate, the panel was not significantly different from that of CA125 alone, combined CA125 with HE4 or ROMA (*P* > 0.05), but was significantly higher than that of HE4 alone (*P* < 0.001) (Table 3). And the positive rate of the model combined with any clinical indicator was significantly higher than that of the model alone or any clinical indicator alone (*P* < 0.001) (Table 3).

**Table 3:** Positive rates of the panel, clinical indicators and the combination of panel and clinical indicators.

Indicator	N	Positive (%)	P*	P**
Panel	198	110 (55.56)		
CA125	134	85 (63.43)	0.366	
HE4	89	29 (32.58)	0.004	
CA125 + HE4	89	53 (59.55)	0.652	
ROMA	87	42 (48.28)	0.533	
Panel + CA125	134	111 (82.84)	< 0.001	< 0.001
Panel + HE4	89	61 (68.54)	< 0.001	< 0.001
Panel + CA125 + HE4	89	73 (82.02)	< 0.001	< 0.001
Panel + ROMA	87	65 (74.71)	< 0.001	< 0.001

*The comparison of positive rates between the panel and other indicators.

**The comparison of positive rates between clinical indicators and the combination of panel and clinical indicators.

## Discussion

Ovarian cancer is a fatal malignancy of the female reproductive system, and has an aggressive tendency to develop from early stage to late stage within 1 year, with one study showing that more than 70% of patients are diagnosed with advanced stage^2^. The prognosis of OC patients is poor, and the 5-year survival rate is only about 40%^30^, while the survival rate of early diagnosed cases is significantly higher, and the 5-year survival rate can reach 90%^3^. For autoantibodies, their properties of stability and early detectability make them an attractive source of serological biomarkers for early detection^8–10^. Since the development of tumors is caused by a variety of abnormalities, it has been shown that the combination of multiple anti-TAA autoantibodies can significantly improve the sensitivity of early tumor diagnosis^31–33^. In cancers that have been studied for a longer period of time, such as lung cancer, combined anti-TAA autoantibodies panels with a considerable diagnostic capability have been produced^34^ and are already being tested in population screening studies^35^. Therefore, this study attempted to explore new anti-TAA autoantibodies as serological markers for OC diagnosis and to establish an optimal diagnostic panel based on them.

In this study, ELISA technology was used to detect the serum anti-TAA autoantibodies of 5 TAAs of interest, among which anti-CFL1, anti-EZR, anti-CYPA and anti-PFN1 showed significant differences between groups in two independent datasets. In previous studies, these four TAAs have been found to be associated with the tumorigenesis and progression of OC. Cofilin 1 (CFL1) is a crucial regulator of actin dynamics and cell migration, and is generally regarded as an accessory in tumor cell invasion and movement^27,36^. The protein expression level of CFL1 was positively correlated with tumor differentiation and gradually increased in normal, benign, borderline and cancerous ovarian tissues, respectively^37^. For patients with epithelial OC, the presence of low CFL1 protein expression is linked to a longer progression-free survival^15^. Ezrin (EZR) is a key membrane cytoskeletal crosslinker and is involved in signal transduction. The study showed that Ezrin protein was overexpressed in OC tissues and cells compared with normal controls, with the highest expression in metastatic tissues and cells, and positively regulates the proliferation, invasiveness and epithelial mesenchymal transformation of ovarian cancer cells^16,38^. Cyclophilin A (CyPA) belongs to the family of immunophilin proteins and is commonly overexpressed in cancer, regulating malignant transformation and metastasis^39^. The research showed that Cyclophilin A may be a potential marker of chemotherapy sensitivity in advanced OC^40^. Another study showed increased expression of CYPA-related peptides in plasma of OC patients compared to healthy controls^17^. Profilin 1 (PFN1) is a member of the actin-binding protein family and has a tissue context-specific role in cell migration and tumor malignancy^41,42^. Proteomics based study has found that PFN1 expression is elevated in OC associated with BRCA1 deficiency^18^.

There are many reasons for the production of anti-TAA autoantibodies, and according to the results of previous studies, abnormal expression may be the most common driver of anti-TAA autoantibodies production^7^. For example, oncoproteins (e.g. c-myc, cyclins and CDK2) are overexpressed in malignant cells, and oncofetal antigens (e.g. IMP1), normally expressed during prenatal development, are re-expressed in malignant transformed cells. Both have been observed to produce anti-TAA autoantibodies in cancer^34,43,44^. According to the Gene Expression Profiling Interactive Analysis (GEPIA) database, in 426 OC tissues and 88 normal ovarian tissues, the mRNA levels of CFL1, CYPA and PNF1 were significantly higher in OC tissues, while the mRNA levels of EZR tended to be higher^45^. The protein expression of TAAs was searched by the Clinical Proteomic Tumor Analysis Consortium (CPTAC), and it was found that compared with 19 normal ovarian tissues, EZR and CFL1 were significantly over-expressed in 84 OC tissues, while CYPA showed an elevated trend^46^. Immunohistochemical data from the Human Protein Atlas (HPA) database showed similar results^47^. The above results may be related to the generation of these anti-TAA autoantibodies. Similar to the results of this study, the anti-TAA autoantibodies of CFL1, EZR, CYPA have been shown certain diagnostic performance in esophageal cancer, pancreatic cancer, and breast cancer^20–24^. Most of the samples included in this study were serous type OC, and only 8 cases of ovarian clear cell carcinoma (OCCC) were known, and NAPSA was often abnormally high expressed in OCCC, which may be the reason for the insignificant difference in anti-TAA autoantibodies^26,48^. Given the ability of machine learning (ML) methods to detect key features from complex data sets, various ML methods have been used in cancer diagnosis to build predictive models and improve the accuracy of decisions^49^. The model constructed by the logistic regression combined with the stepwise forward method was selected as the optimal model, which showed good value in the diagnosis of OC.

At present, CA125 and HE4 are the best serological markers for the diagnosis of OC, and their expression is independent of each other to a certain extent^50,51^. The combination of CA125 and HE4, particularly the ROMA formula that incorporates menopausal status parameters, has been shown to have a more accurate diagnostic value than any single indicator^52,53^. Previous studies have shown that multiplex detection containing multiple types of biomarkers can improve diagnostic value, such as monoclonal antibody plus CA125 can better differentiate OC compared to CA125 alone^54,55^. Therefore, this study compared the positive rate of generated anti-TAA autoantibodies panels with clinical indicators in OC patients. Our results show that the positive rate of the panel combined with any clinical indicator was significantly higher than that of the panel alone or any clinical indicator alone, and the panel could identify 53.06% (26/49) CA125-negative patients, 55.88% (38/68) HE4-negative patients, 56.41% (22/39) of patients with CA125 and HE4 were all negative, and 51.11% (23/45) of ROMA-negative patients. Given that the anti-TAA autoantibody enzyme-linked immunosorbent assay is easily translated into a clinical chemistry platform, the panel we constructed can be easily added to the existing screening biomarkers CA125 and HE4. Taken together, the panel we constructed has the potential to complement existing clinical indicators for clinical implementation.

This study is the first to investigate the application of anti-CFL1, anti-EZR, anti-CYPA, anti-PFN1 and anti-NAPSA in the diagnosis of OC. Compared to previous studies on OC and anti-TAA autoantibodies, this study has the advantages of a larger sample size, specific detection in the group of benign ovarian diseases, and comparison with existing clinical indicators. In addition, the design of two different sets and samples from different hospitals makes our results more reliable. However, this study also has some limitations. It is a retrospective case–control study, and prospective specimens cannot be collected from the cohort; the specificity of the indicators was not detected in other cancers, and they may be widely expressed in various cancer types, resulting in reduced panel specificity. In the long run, further validation of anti-TAA autoantibodies signatures in a large-scale prospective study and serums from other cancer patients is necessary.

In conclusion, this study demonstrated the existence of the above five anti-TAAs antibodies and found some new anti-TAA autoantibodies that can be used in OC diagnosis. The optimal combination of the three anti-TAA autoantibodies can serve as a good tool for diagnosis of OC and has the potential to be a complementary biomarker for CA125 and HE4 to improve the diagnostic value of OC.

### Supplementary Information

Supplementary Tables.

## Data Availability

The raw datasets used and/or analysed during the current study are available from the corresponding author on reasonable request.
