# Supplementary material for: Serum anti-CFL1, anti-EZR, and anti-CYPA autoantibody as diagnostic markers in ovarian cancer
Source: Sci Rep. 2024 Apr 29;14:9757. doi: 10.1038/s41598-024-60544-2 (PMC11058243; doi:10.1038/s41598-024-60544-2)
Supplement: Supplementary file 1 — Supplementary Tables. [file 41598_2024_60544_MOESM1_ESM.pdf]

**Supplementary table 1** Five tumor-associated autoantibodies (TAAbs) levels between ovarian cancer (OC) and normal control (NC) groups in discovery and validation cohorts.

| anti-TAA autoantibodies | Discovery cohort | | | | Validation cohort | | | |
|---|---|---|---|---|---|---|---|---|
| | OC (n=70) | NC (n=70) | *Z* | *P* | OC (n=128) | NC (n=128) | *Z* | *P* |
| anti-NAPSA | 0.349(0.257-0.419)* | 0.325(0.243-0.381) | 0.796 | 0.426 | - | - | - | - |
| anti-CFL1 | 0.242(0.136-0.452) | 0.139(0.090-0.261) | 3.724 | ＜0.001 | 0.226(0.118-0.411) | 0.128(0.085-0.215) | 5.004 | ＜0.001 |
| anti-EZR | 0.351(0.250-0.434) | 0.270(0.204-0.335) | 3.493 | ＜0.001 | 0.314(0.239-0.410) | 0.250(0.189-0.323) | 4.553 | ＜0.001 |
| anti-CYPA | 0.228(0.143-0.304) | 0.148(0.119-0.211) | 3.838 | ＜0.001 | 0.197(0.147-0.296) | 0.151(0.111-0.201) | 5.146 | ＜0.001 |
| anti-PFN1 | 0.150(0.102-0.233) | 0.124(0.096-0.164) | 2.317 | 0.020 | 0.143(0.102-0.207) | 0.116(0.092-0.148) | 3.556 | ＜0.001 |

Note: The calculated value is the optical density. OC, Ovarian cancer; NC, normal control; BD, benign disease.

*Median (25 percentile to 75 percentile).

**Supplementary table 2** The diagnostic value of tumor-associated autoantibodies (TAAbs) for identifying OC from normal control (NC).

| anti-TAA autoantibodies | Positive(%) | | χ2 | *P* | Se(%) | Sp(%) | YI | Accuracy(%) | PPV(%) | NPV(%) | +LR | -LR |
|---|---|---|---|---|---|---|---|---|---|---|---|---|
| | OC | NC | | | | | | | | | | |
| Discovery cohort | | | | | | | | | | | | |
| anti-NAPSA | 10(14.29) | 3(4.29) | 4.16 | 0.04 | 14.29 | 95.71 | 0.10 | 55.00 | 76.91 | 52.76 | 3.33 | 0.90 |
| anti-CFL1 | 26(37.14) | 4(5.71) | 20.53 | <0.01 | 37.14 | 94.29 | 0.31 | 65.72 | 86.67 | 60.00 | 6.50 | 0.67 |
| anti-EZR | 20(28.57) | 7(10.00) | 7.76 | <0.01 | 28.57 | 90.00 | 0.19 | 59.29 | 74.07 | 55.75 | 2.86 | 0.79 |
| anti-CYPA | 23(32.86) | 7(10.00) | 10.86 | <0.01 | 32.86 | 90.00 | 0.23 | 61.43 | 76.67 | 57.27 | 3.29 | 0.75 |
| anti-PFN1 | 18(25.71) | 4(5.71) | 10.57 | <0.01 | 25.71 | 94.29 | 0.20 | 60.00 | 81.83 | 55.93 | 4.50 | 0.79 |
| Validation cohort | | | | | | | | | | | | |
| anti-CFL1 | 43(33.59) | 11(8.59) | 24.03 | <0.01 | 33.59 | 91.41 | 0.25 | 62.50 | 79.63 | 57.92 | 3.91 | 0.73 |
| anti-EZR | 24(18.75) | 11(8.59) | 5.59 | <0.01 | 18.75 | 91.41 | 0.10 | 55.08 | 68.58 | 52.94 | 2.18 | 0.89 |
| anti-CYPA | 39(30.47) | 9(7.03) | 23.08 | <0.01 | 30.47 | 92.97 | 0.23 | 61.72 | 81.25 | 57.21 | 4.33 | 0.75 |
| anti-PFN1 | 45(35.16) | 12(9.37) | 24.48 | <0.01 | 35.16 | 90.63 | 0.26 | 62.90 | 78.96 | 58.29 | 3.75 | 0.72 |

Note: The cut-off value was considered as the maximum Youden index at the point of more than 90% specificity.

FNR, false-negative rate; FPR, false-positive rate; NPV, negative predictive value; PPV, positive predictive value; Se, sensitivity; Sp, specificity; YI, Youden index

**Supplementary table 3** The diagnostic value of different machine learning models after 10 times 10-fold cross-validation in the validation cohort.

| Methods | TAAbs | AUC | Se(%) | Sp(%) |
|---|---|---|---|---|
| random forest | 4* | 0.694 | 61.83 | 60.21 |
| support vector machine | 4 | 0.726 | 69.31 | 64.94 |
| gradient boosting decision tree | 4 | 0.729 | 68.20 | 61.63 |
| Naive Bayes | 4 | 0.749 | 82.29 | 51.60 |
| neural network | 4 | 0.753 | 71.58 | 62.93 |
| forward logistic regression | 3** | 0.759 | 73.15 | 62.78 |

*4：CFL1+EZR+CYPA+PFN1

**3：CFL1+EZR+CYPA

**Supplementary table 4** The diagnostic value of tumor-associated autoantibodies (TAAbs) for identifying OC from normal control (NC).

| anti-TAA autoantibodies | Positive(%) | | $\chi 2$ | *P* | Se(%) | Sp(%) | YI | Accuracy(%) | PPV(%) | NPV(%) | +LR | -LR |
|---|---|---|---|---|---|---|---|---|---|---|---|---|
| | OC | NC | | | | | | | | | | |
| BD validation cohort (OC vs.BD) | | | | | | | | | | | | |
| anti-CFL1 | 17(20.99) | 7(8.64) | 4.89 | 0.027 | 20.99 | 91.36 | 0.12 | 56.18 | 70.84 | 53.62 | 2.43 | 0.86 |
| anti-EZR | 11(13.58) | 4(4.94) | 3.60 | 0.058 | 13.58 | 95.06 | 0.09 | 54.32 | 73.33 | 52.38 | 2.75 | 0.91 |
| anti-CYPA | 21(25.93) | 7(8.64) | 8.46 | 0.004 | 25.93 | 91.36 | 0.17 | 58.65 | 75.01 | 55.23 | 3.00 | 0.81 |
| BD validation cohort (OC vs.BD+NC) | | | | | | | | | | | | |
| anti-CFL1 | 28(34.57) | 14(91.36) | 25.39 | <0.001 | 34.57 | 91.36 | 0.26 | 72.43 | 66.67 | 73.63 | 4.00 | 0.72 |
| anti-EZR | 15(18.52) | 15(90.74) | 4.278 | 0.039 | 18.52 | 90.74 | 0.09 | 66.67 | 50.00 | 69.01 | 2.00 | 0.90 |
| anti-CYPA | 26(32.10) | 15(90.74) | 20.08 | <0.001 | 32.10 | 90.74 | 0.23 | 71.19 | 63.41 | 72.77 | 3.47 | 0.75 |

Note: The cut-off value was considered as the maximum Youden index at the point of more than 90% specificity.

FNR, false-negative rate; FPR, false-positive rate; NPV, negative predictive value; PPV, positive predictive value; Se, sensitivity; Sp, specificity; YI, Youden index

**Supplementary table 5** The diagnostic value of the panel with three tumor-associated autoantibodies for OC subgroups

| Group | N | Positive (%) | *P** | *P*** | Sensitivity (%) | Specificity (%) | YI | Accuracy (%) | PPV (%) | NPV (%) | +LR | -LR |
|---|---|---|---|---|---|---|---|---|---|---|---|---|
| all stage | 198 | 110(55.56) | | | 55.56 | 81.31 | 0.37 | 68.44 | 74.83 | 64.66 | 2.97 | 0.55 |
| Early stage (I-II) | 50 | 27(54.00) | 0.999 | 0.995 | 54.00 | 82.32 | 0.36 | 68.16 | 43.54 | 87.63 | 3.05 | 0.56 |
| Late stage (III-IV) | 128 | 73(57.03) | 0.992 | | 57.03 | 81.31 | 0.38 | 69.17 | 66.36 | 74.54 | 3.05 | 0.53 |
| age<50 | 67 | 34(50.75) | 0.227 | 0.073 | 50.75 | 81.31 | 0.32 | 66.03 | 47.88 | 82.99 | 2.72 | 0.61 |
| age≥50 | 131 | 76(58.02) | 0.413 | | 58.02 | 81.31 | 0.39 | 69.67 | 67.25 | 74.54 | 3.10 | 0.52 |
| Epithelial tumor | 161 | 86(53.42) | 0.834 | 0.574 | 53.42 | 81.31 | 0.35 | 67.37 | 69.92 | 68.22 | 2.86 | 0.57 |
| Other histological types | 17 | 9(52.94) | 0.642 | | 52.94 | 96.97 | 0.50 | 74.96 | 60.00 | 96.00 | 17.47 | 0.49 |
| Lymph node metastasis (-) | 58 | 29(50.00) | 0.737 | 0.797 | 50.00 | 82.32 | 0.32 | 66.16 | 45.31 | 84.90 | 2.83 | 0.61 |
| Lymph node metastasis (+) | 119 | 67(56.30) | 0.938 | | 56.30 | 81.31 | 0.38 | 68.81 | 64.42 | 75.59 | 3.01 | 0.54 |
| Distant metastasis (-) | 139 | 76(54.68) | 0.854 | 0.704 | 54.68 | 81.31 | 0.36 | 68.00 | 67.25 | 71.88 | 2.93 | 0.56 |
| Distant metastasis (+) | 44 | 25(56.82) | 0.799 | | 56.82 | 81.31 | 0.38 | 69.07 | 40.32 | 89.44 | 3.04 | 0.53 |
| Menopause (-) | 31 | 17(54.84) | 0.582 | 0.610 | 54.84 | 81.31 | 0.36 | 68.08 | 31.48 | 92.00 | 2.93 | 0.56 |
| Menopause (+) | 157 | 85(54.14) | 0.956 | | 54.14 | 81.31 | 0.35 | 67.73 | 69.67 | 69.10 | 2.90 | 0.56 |
| CA125 (-) | 49 | 26(53.06) | 0.879 | 0.654 | 53.06 | 82.32 | 0.35 | 67.69 | 42.62 | 87.63 | 3.00 | 0.57 |
| CA125 (+) | 85 | 51(60.00) | 0.685 | | 60.00 | 81.31 | 0.41 | 70.66 | 57.95 | 82.56 | 3.21 | 0.49 |
| HE4 (-) | 60 | 32(53.33) | 0.440 | 0.978 | 53.33 | 81.31 | 0.35 | 67.32 | 50.22 | 83.13 | 2.85 | 0.57 |
| HE4 (+) | 29 | 17(58.62) | 0.892 | | 58.62 | 81.82 | 0.40 | 70.22 | 24.57 | 95.14 | 3.22 | 0.51 |
| CA125+HE4 (-) | 36 | 20(55.56) | 0.813 | 0.703 | 55.56 | 82.32 | 0.38 | 68.94 | 38.83 | 90.17 | 3.14 | 0.54 |
| CA125+HE4 (+) | 53 | 29(54.72) | 0.561 | | 54.72 | 81.31 | 0.36 | 68.02 | 42.51 | 87.67 | 2.93 | 0.56 |
| ROMA (-) | 45 | 23(51.11) | 0.453 | 0.726 | 51.11 | 81.31 | 0.32 | 66.21 | 38.33 | 87.98 | 2.73 | 0.60 |
| ROMA (+) | 42 | 24(57.14) | 0.747 | | 57.14 | 81.82 | 0.39 | 69.48 | 40.00 | 90.00 | 3.14 | 0.52 |

Note: The control group was 198 normal controls.

*means comparison between each subgroup and all stages using DeLong's test.

**means comparison between subgroup of the row and subgroup of the next row with the method of DeLong's test.

FNR, false-negative rate; FPR, false-positive rate; NPV, negative predictive value; PPV, positive predictive value; Se, sensitivity; Sp, specificity; YI, Youden index
